# Down-Regulation of CD9 by Methylation Decreased Bortezomib Sensitivity in Multiple Myeloma

**DOI:** 10.1371/journal.pone.0095765

**Published:** 2014-05-02

**Authors:** Xiaotong Hu, Han Xuan, Huaping Du, Hao Jiang, Jinwen Huang

**Affiliations:** 1 Biomedical Research Center, Sir Run Run Shaw Hospital, Zhejiang University and Key Laboratory of Biotherapy of Zhejiang Province, Hangzhou, China; 2 Department of Hematology, Sir Run Run Shaw Hospital, Zhejiang University, Hangzhou, China; Florida International University, United States of America

## Abstract

Bortezomib therapy has been proven successful for the treatment of relapsed and/or refractory multiple myeloma (MM). However, both intrinsic and acquired resistance has already been observed. In this study, we explored the relationship between CD9 expression and bortezomib sensitivity in MM. We found that down-regulation of CD9 by methylation decreased bortezomib sensitivity in multiple myeloma. CD9 expression obviously increased bortezomib sensitivity through inducing apoptosis, significantly inhibiting U266 cells' adhesion to HS-5 and primary bone marrow stromal cells, but increasing U266 cells' adhesion to fibronectin. CD9 expression also significantly inhibited U266 cell migration. The mechanisms may include: the endoplasmic reticulum stress pathway, cell adhesion related signaling pathway and osteoclast differentiation related signaling pathway. Combination therapy with de-methylation reagent 5-Aza-2-deoxycytidine may prove useful to the development of novel strategies for the treatment of bortezomib-resistant MM patients.

## Introduction

Multiple myeloma (MM) is an incurable plasmatic neoplasm among the hematological malignancies. Despite great advances in understanding the molecular pathogenesis of MM and the development of promising new therapies, only 25–35% of patients respond to therapies in the relapsed and refractory setting. Bortezomib (PS-341; Velcade) therapy has proven successful for the treatment of relapsed and/or refractory multiple myeloma. However, both intrinsic and acquired resistance has already been observed in MM patients [1]. This prompts a growing interest in understanding its mechanisms of bortezomib treatment and resistance. Molecular studies have identified many potential therapeutic targets of bortezomib. Bortezomib can directly inhibits the proliferation of myeloma cells, induces their apoptosis, and abrogates paracrine tumor growth through alteration of interactions of myeloma and stromal cells. It is well established that the physical interaction between MM cells and the bone marrow (BM) microenvironment plays a crucial role in MM pathogenesis and drug resistance. Direct interaction between MM cells and BM cells activates pleiotropic signaling pathways that mediate growth, survival, migration of MM cells and drug resistance, as well as angiogenesis and BM osteoclastogenesis.

CD9, a member of tetraspanin family, was found to be down-regulated in relapsed MM cells after treatment with bortezomib (Li X *et al*, unpublished data). Tetraspanins span the membrane four times and accumulate in membrane microdomains, distinct from lipid rafts. More than 20 family members are reported for mammals, including CD9, CD63, CD81, CD82 and CD151. They associate with other proteins in either a direct or indirect fashion. This entire complex of interactions has been termed the ‘‘tetraspanin web’’ [2]–[4]. CD9, one of the most characterized members of the tetraspanins, was initially reported to be expressed in pre-B cells and platelets [5], [6], but further studies have revealed that it is expressed in a wide variety of hematopoietic and non-hematopoietic cells [7]–[10]. It interacts with other cell adhesion molecules and plays on the role of the organizer in tetraspanins net [11]. It has been implicated in various biological functions, including cell adhesion, motility, metastasis, growth, signal transduction, differentiation, and sperm-egg fusion [2], [12], [13].

The implication of CD9 in cancer has received much attention. An inverse correlation between its expression in primary tumors and the metastatic potential and patient survival rate has been established in various carcinomas [14]–. For example, the expression of CD9 significantly decreased in metastatic breast cancer, colonic cancer and prostate cancer cells than in primary tumor cells, while the high expression of CD9 could weaken migration ability of various kinds of tumor cells [18], [19]. For oral squamous cell carcinomas, the low expression of CD9 indicated the late stage of tumor development and the low survival rate [20]. Restore expression of CD9 in small cell lung cancer cells can significantly inhibit the proliferation and migration of tumor cells [21]. All of these results show that the down-regulation of CD9 plays an important role in the development of tumor.

In this study, we checked (1) the CD9 expression and their methylation control mechanism in MM cell lines and primary cases, (2) whether CD9 expression has a special relationship with bortezomib sensitivity in MM and (3) whether a combination of low dose de-methylation reagent 5-Aza-2-deoxycytidine (5-Aza) and bortezomib can overcome bortezomib resistance in MM.

## Materials and Methods

### Cell culture

The human multiple myeloma cell lines U266, NCI-H929, RPMI8226, MM.1S, OPM 2 and HS-5 were purchased from the American Type Culture Collection (ATCC, Manassas, VA, USA). The cells were maintained in RPMI 1640 medium supplemented with 15% fetal bovine serum, 100 units/mL penicillin, and 100 mg/mL streptomycin in a humidified atmosphere with 5% CO_2_ at 37°C. Fresh bone marrow samples from newly diagnosed patients with MM without any treatment were obtained at the Sir Run Run Shaw Hospital, Zhejiang University. Written informed consent for the use of the tissues was obtained from all patients and the study was approved by the Institute Research Ethics Committee of Sir Run Run Shaw Hospital, Zhejiang University.

### Isolation of primary myeloma and stromal cells from MM patients

Fresh CD138 positive MM cells were isolated from the mononuclear fraction using anti-human CD138 microbeads (Miltenyi Biotec, Auburn, CA) by AutoMACS according to manufacturer's instructions. Purity of plasma cells ranged between 60% and 90%, based on fluorescence-activated cell sorting (FACS) analysis of CD138 expression (FACSCaliber; BD Biosciences, San Jose, CA).

Bone marrow stromal cells (BMSC) were prepared by seeding the remaining mononuclear cells after CD138-positive selection. The cells were seeded in DMEM complete culture medium (10% fetal bovine serum, 100 units/ml penicillin, 100 mg/ml streptomycin, and 2 mM L-glutamin, Gibco). After 3 days of culture, non-adherent cells were removed, and the remaining cells were expanded and split after about 10 days.

### Co-culture of MM cells with HS-5 or primary BMSC cells

HS-5 or primary BMSC cells were plated in 6-wells at 10^5^ cells/well. Upon confluence, each group of U266 cells (0.5×10^6^/well) were added. Drugs were added at the concentrations indicated. The cells were cultivated in a total volume of 200 ml/well.

### Construction of the lentivirus-encoding CD9

To construct the lentivirus-encoding CD9 (lenti-CD9) plasmid (pLenO-DCE-Puro-CD9), the cDNA-encoding CD9 (NM_001769.3) was synthesized and cloned into the EcoRI and BamHI restriction endonuclease sites of the pLenO-DCE-Puro vector (Invabio, Shanghai, China), a mammalian expression vector containing green fluorescent protein (GFP) and puromycin resistance genes. The DH5a Escherichia coli strain was used for amplification of lenti-CD9. After the correct sequence was confirmed by sequencing, lenti-CD9 was introduced into 293T cells using four-plasmid co-transfection with pRSV-Rev (packaging helper plasmid), pMDLg/pRRE, pMD2G and pLenO-DCE-Puro-CD9 (transfer vector). A lentivirus containing the empty transfer vector pLenO-DCE-Puro (lenti-GFP) was also generated. At 48 h after infection, the number of GFP-positive cells was measured using FACS, and the titer was determined.

### Generation of CD9 stably transfected U266 cells

Briefly, when the U266 cells reached 50-60% confluence, the medium was removed and washed twice with PBS. Polybrene was used to increase the infection rate, and the infection was performed with lenti-CD9 and lenti-GFP according to the manufacturer's instructions. At 2 days post-infection, the percentage of GFP-positive U266 cells was determined using a fluorescence microscope to evaluate the infectivity. GFP-positive cells were selected by flow cytometry.

### RNA extraction, cDNA synthesis, and semi-quantitative RT-PCR

Total RNA was extracted from cell pellets by Trizol (Invitrogen, Carlsbad, CA, USA) and reverse transcribed into cDNA using MultiScribe Reverse Transcriptase (Applied Biosystems, Foster city, CA, USA) according to the manufacturer's instructions. The target gene expression was determined by semi-quantitative RT-PCR using specific primers of CD9 gene (Table S1). GAPDH was served as an internal control for total cDNA content. Samples were amplified using the ABI 7500 Real-Time PCR Systems (Applied Biosystems, Foster City, CA, USA).

### 5-Aza-2-deoxycytidine (5-Aza) treatment

Cells were treated with 10 µM de-methylating agent, 5-Aza (Sigma-Aldrich, St Louis, MO, USA) for 3 days. After treatment the cells were harvested for DNA and RNA extraction.

### Bisulphite treatment and promoter methylation analysis

Bisulphite modification of DNA, Methylation Specific PCR (MSP) and Bisulphate Genome Sequencing (BGS) were carried out as described previously [22], [23]. MSP and BGS primers are listed in Table S1.

### Cell viability assay

Cells were seeded at 1×10^5^cells/100 ml/well in 96-well plates and exposed to various concentrations of bortezomib for 24 hours. Cell viability was quantified using the CellTiter 96 AQueous Non-Radioactive Cell Proliferation Assay (Promega, Madison, WI, USA). Each well was treated with MTS for 1 to 4 hours, after which absorbance at 490 nm was recorded using a 96-well plate reader. The quantity of formazan product as measured is directly proportional to the number of living cells. The results were derived from three independent experiments performed in triplicate. Calculation of the 50% inhibitory concentration (IC50) was done using SPSS 16.0 software.

### Cell cycle analysis

Each group of cells were harvested, fixed with cold 70% ethanol, and suspended in 50 mg/ml PI (Sigma-Aldrich, St. Louis, MO, USA) with the addition of 0.1 mg/mL RNase A. After incubation at 37°C for 30 mins, cell cycle flow cytometry analysis was performed by means of fluorescence activated cell sorting (FACS).

### Apoptotic assays

Each group of cells was harvested by pipetting, washed with phosphate buffered saline, and stained with PE-conjugated annexin-V (annexin-V/PE) (Beyotime, Nanjing, China). Cell apoptosis was judged by annexin-V reactivity in GFP positive populations using flow cytometer.

### Cell adhesion assay

After 48-hour co-culture of U266 cells with primary BMSC, HS-5 cells or Fibronectin were performed, the cell adhesion assay was carried out using Vybrant^@^ Cell Adhesion Assay Kit (Invitrogen, Carlsbad, CA, USA) following the manufacturer's recommendations. We determined the percentage of adhesion by dividing the corrected (background subtracted) fluorescence of adherent cells by the total corrected fluorescence of cells added to each well.

### Cell migration assay

Each group of U266 cells was serum starved overnight and then resuspended in 70 uL of RPMI1640 medium/0.5% fetal calf serum. Cell migration was conducted in 24-well, 6.5 mm internal diameter transwell cluster plates (Corning Costar; Cambridge, MA). Briefly, cells (1×10^5^/250 µl) were loaded onto polycarbonate membranes (8 µm pore size) separating 2 chambers of a transwell. Medium/0.1% FCS (500 µl) was added to the lower chamber of the transwell cluster plates. After 24 hours, cells migrating into the lower chamber were counted under a light microscope. The experiment was performed three times.

### Methylcellulose colony formation assay

Methylcellulose media consisted of RPMI 1640 containing 1.1% methylcellulose (Aqua Solutions, Deer Park, TX), 30% fetal bovine serum, 100 units/ml penicillin, 100 mg/ml streptomycin, and puromycin at the appropriate concentration for each cell line. Cells were plated at a density of 500 cells/ml in 1 ml volume in humidified 24-well plates. Colonies were counted between 10 and 15 days after plating.

### Western blot analysis

Proteins were separated from cell lysates by SDS-PAGE, transferred to nitrocellulose, and probed with FAK, P-397 FAK, P-925 FAK, P-576/577 FAK, TRB3, CHOP, PERK, Ero1 La, IRE1a, PDI, Calnexin antibodys (Cell Signaling Technology, Beverly, MA, USA). The blots were developed by enhanced chemiluminescence (ECL).

### Microarray and data analysis

Total RNA from 6 sample (3 U266-CD9 and 3 U266-GFP) was quantified by the NanoDrop ND-1000 and RNA integrity was assessed by standard denaturing agarose gel electrophoresis and then used for labeling and array hybridization. The labeled cDNA samples were submitted to Roche NimbleGen and hybridized to Human 12×135K Gene Expression Arrays. The results were scanned using a Agilent Scanner G2505C and imported into NimbleScan software (version 2.5) for grid alignment and expression data analysis. Differentially expressed genes with statistical significance (Fold Change ≥ 2.0, P-value ≤ 0.05) were shown by Volcano Plot filtering (Figure S1). Pathway Analysis and GO analysis were applied to determine the roles of these differentially expressed genes played in these biological pathways or GO terms. The results obtained were submitted to Gene Expression Omnibus (GEO) database and the accession number is GSE55818.

### Statistical analysis

Results are expressed as values of mean ± standard deviation (SD). Statistical analysis was performed using SPSS 16.0 for Windows (SPSS Inc., Chicago, IL, USA) and Student's t-test was used. p values less than 0.05 were considered statistically significant.

## Results

During the study of MM cell lines resistant to bortezomib treatment, we found a most down-regulated gene was a tetraspanin family protein, CD9. CD9 expression was also found to be significantly higher in the patients sensitive to bortezomib than in the patients resistant to bortezomib (Li X *et al*, unpublished data).

**Figure 1:**
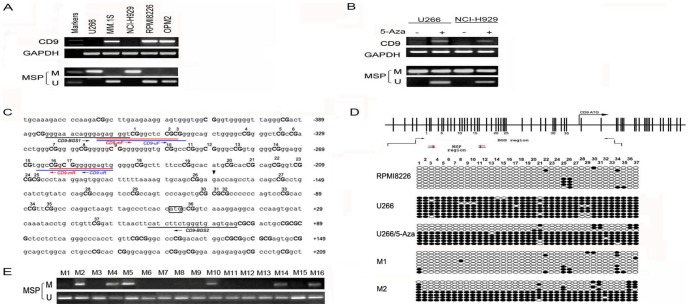
Analyses of methylation status of CD9 in multiple myeloma cell lines and cases. (A) Silencing of CD9 by promoter methylation in cell lines detected by semi-quantitative RT-PCR, with GAPDH as a control. M: methylated; U: unmethylated. (B) CD9 expression reserved with de-methylation reagent 5-Aza in U266 and NCI-H929. (C) Sequence of CD9 CGI with locations of the 37 CpG sites analysed and primers used. Methylation-specific PCR (MSP) and bisulfite genomic sequencing (BGS) regions are also shown. (D) Vertical lines indicate individual CpG sites. Cloned BGS-PCR products were sequenced and each clone was shown as an individual row, representing a single allele of the CGI. Filled circle, methylated; open circle, unmethylated. (E) Analysis of CD9 methylation in primary cases by MSP. M, methylated; U, unmethylated.

Then we evaluated the bortezomib IC50 in several myeloma cell lines. Our results suggested that in MM cells sensitive to bortezomib treatment, IC50 values being 5 nmol/L for RPMI-8226 cells and 6.2 nmol/L for MM.1S cells. However in MM cells resistant to bortezomib treatment, IC50 values were significantly higher, being 20.9 nmol/L for U266 cells and 18.5 nmol/L for NCI-H929 cells. Then we examined CD9 expression in these cell lines by semi-quantitative RT-PCR. CD9 expressed in bortezomib sensitive RPMI-8226 and MM.1S cells but silenced in bortezomib resistant U266 and NCI-H929 cells (Figure 1A).

Since CD9 gene has CpG island (CGI) in the promoter region, we designed methylation-specific PCR (MSP) primers to analyse its methylation status in these cell lines. The CD9 CGI was methylated in U266 and NCI-H929 cells with silenced CD9 expression (Figure 1A). Moreover, CD9 expression was significantly induced after 5-Aza treatment in these cells (Figure 1B). We further examined the detailed methylation profiles of CD9 CGI by bisulfite genomic sequencing (BGS) analysis of 37 CpG sites, including those CpG sites analysed by MSP (Figure 1C). Densely methylated CpG sites were detected in cells with no CD9 expression, representative results are shown in Figure 1D. Both MSP and BGS showed that the CD9 CGI was dramatically demethylated after 5-Aza treatment, showing a direct link between CGI methylation and CD9 silencing (Figure 1B and 1D). We also analysed CD9 methylation status in 16 primary MM cases. CD9 methylation was detectedin 37.5% (6/16) of tumors (Figure 1E).

**Figure 2:**
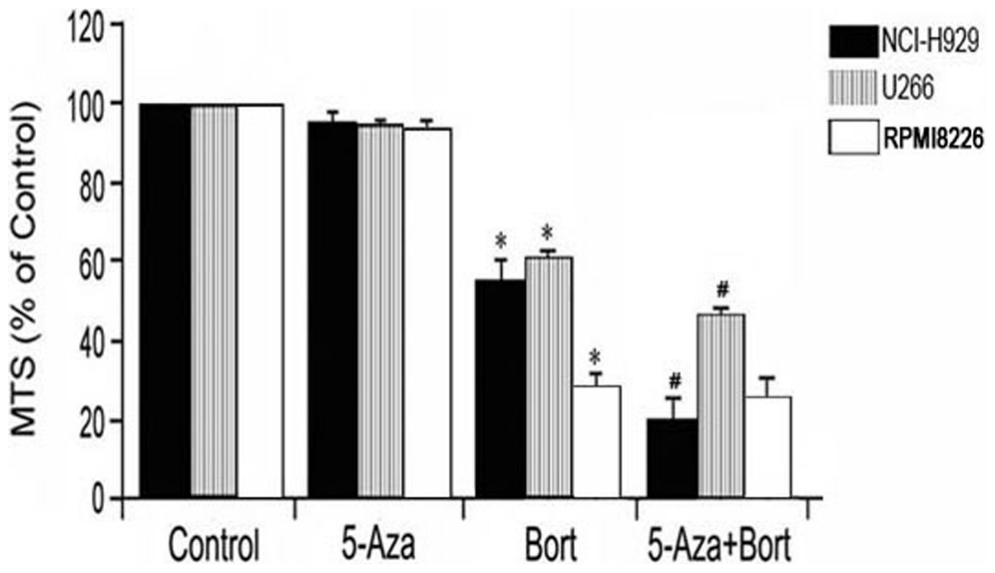
Synergy interaction of 5-Aza and bortezomib in MM cell lines. After pretreated with 5-Aza for 72 hours and then bortezomib, the sensitivity to bortezomib in U266 and NCI-H929 raised significantly while the bortezomib sentitive RPMI8226 raised a little bit.

By MTS analysis we also found that after pretreatment of 5-Aza, the sensitivity to bortezomib in U266 and NCI-H929 raised significantly while the bortezomib sentitive RPMI8226 raised a little bit (Figure 2), suggesting the synergistic interaction of 5-Aza and bortezomib inhibition in multiple myeloma may due to CD9 expression reversion.

**Figure 3:**
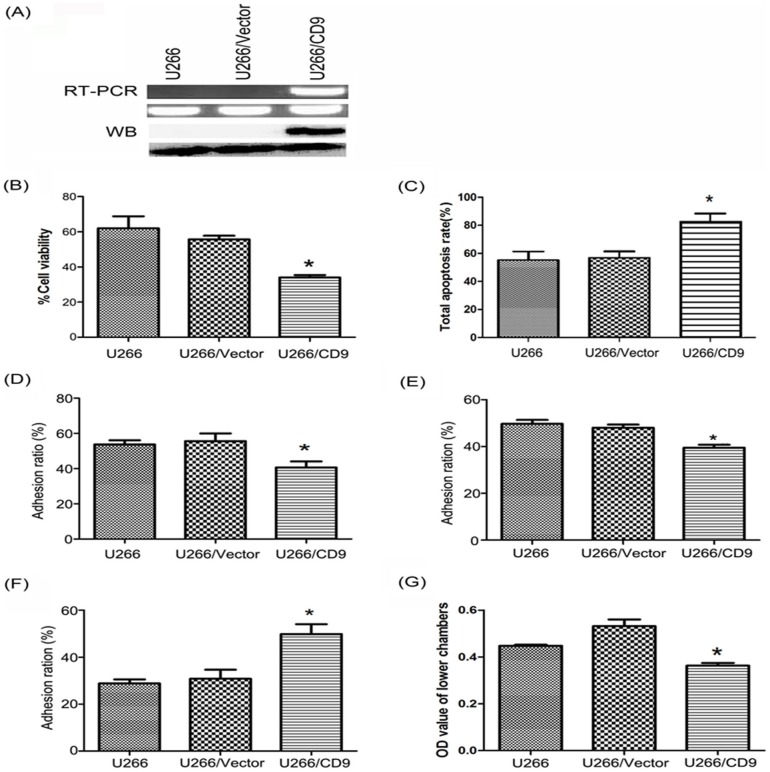
CD9 stablly transfected U266 cells construction and the effects of CD9 expression in U266 cell viability, apoptosis, adhesion and migration. (A) CD9 expression was confirmed by RT-PCR and western blot analysis. (B) CD9 expression obviously increased the bortezomib sensitivity compared to controls, *: p<0.05. (C) Bortezomib significantly promoted the apoptosis of U266/CD9 cells. *: p<0.05. (D) CD9 expression significantly inhibited U266 cells adhesion to bone marrow stromal cells HS-5. *: p<0.05. (E) CD9 expression significantly inhibited U266 cells' adhesion to the primary bone marrow stromal cells. *: p<0.05. (F) CD9 expression significantly increased U266 cells' adhesion to fibronectin. *: p<0.05. (G) CD9 expression significantly inhibited U266 cell migration. *: p<0.05.

In order to study the expression of CD9 and its corresponding cell behavior changes in myeloma cells and their microenvironment, we transfected CD9 in U266 cells and selected the stably transfected cells by flow sorting. CD9 expression was confirmed by RT-PCR and western blot analysis (Figure 3A).

Cell viability results showed that CD9 expression could obviously increase the drug sensitivity (Figure 3B). The main reason may be because the apoptosis ability had improved significantly (Figure 3C). We also tested the drug action on cell autophagy, there was no significant difference between groups of cells (data not shown).

**Figure 4:**
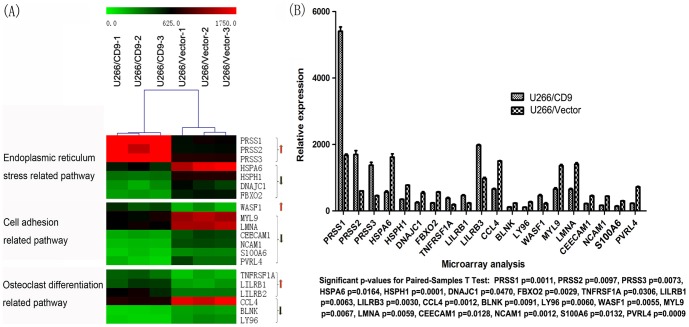
The differentially expressed genes in U266/CD9 and U266/Vector cells tested by genome-wide expression profile chip experiments and the analysis of related signaling pathways. CD9 expression mainly affected three important signaling pathways in MM. They are the endoplasmic reticulum stress pathway, cell adhesion related signaling pathways and osteoclast differentiation related signaling pathway, respectively (A). The degree of differentially expressed genes and p values were also analysed (B).

In order to detect CD9's influence on the biological functions of cells in the bone marrow microenvironment, we mainly studied the effects of CD9 on U266 cell's clone formation ability, cell cycle arrest, cell adhesion and migration, which may be the possible mechanisms of bortezomib sensitivity in MM involving tetraspanins. CD9 did not affect the cell clone formation ability, cell cycle and the major cytokines (VEGF, IL-6 and IGF-1) secreted (data not shown). However, CD9 expression significantly affected the cell adhesion and migration ability. Ectopic expression of CD9 significantly inhibited U266 cells' adhesion to HS-5 (Figure 3D) and primary bone marrow stromal cells (Figure 3E), but CD9 expression increased U266 cells' adhesion to Fibronectin (Figure 3F). CD9 expression also significantly inhibited U266 cell migration (Figure 4G).

In order to study the molecular mechanisms of CD9's effects on bortezomib sensitivity in multiple myeloma, we did the genome-wide expression profile microarray to detect the differentially expressed genes and explore the molecular signaling pathways affected by CD9 expression. We found that CD9 expression mainly affected the following three important signaling pathways respectively: the endoplasmic reticulum stress pathway, cell adhesion related signaling pathway and osteoclast differentiation related signaling pathway (Figure 4).

**Figure 5:**
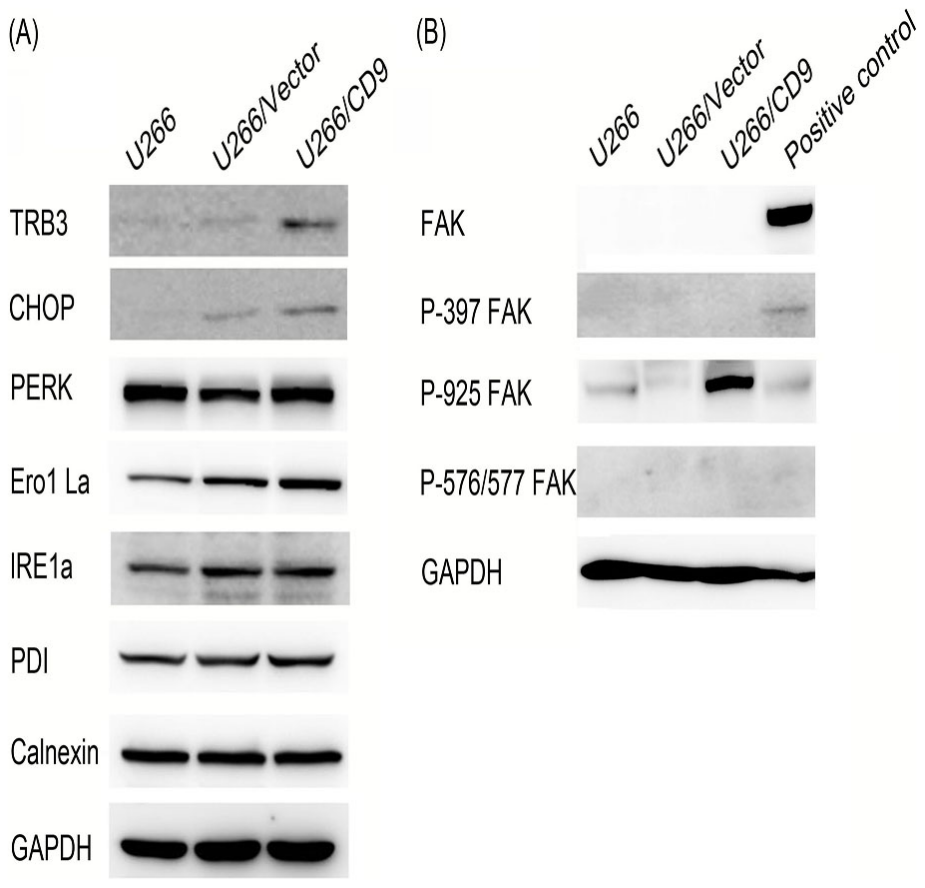
Several key ER Stress related protein and expression of FAK was analyzed by western blotting.

In addition to the differentially expressed genes shown by microarray, we futher checked protein expression level of several key genes involving ER stress pathway. The results showed that TRB3 and CHOP expressed higher in CD9 transfected U266 cells (Figure 5A).

Moreover, though FAK is not the differentially expressed gene detected by microarray, we checked some tyrosine-phosphorylated FAK protein expression using western blot. The results showed that only Phospho-FAK (Tyr925) expressed in cells and significantly increased in CD9 transfected U266 cells (Figure 5B).

## Discussion

In this study, we identified that a tetraspanin family proteins, CD9 was significantly down-regulated and confered bortezomib resistance in MM. CD9 is constitutively expressed on a subpopulation of B cells [24], [25]. Membranal tetraspanins are often inversely correlated with cancer prognosis and metastasis, however mutations were unidentified hitherto [26], [27]. Their promoter characteristics and frequent down-regulation conform to transcriptional silencing by chromatin remodeling. We also found that down-regulation of CD9 due to promter hypermethylation in MM, consistant with previous studies [28]–[30]. Reverse expression of CD9 in the cell lines following de-methylation reagent 5-Aza treatment confirmed the mechanistic significance of methylation to its regulation. After pre-treatment of 5-Aza, the sensitivity to bortezomib in U266 and NCI-H929 raised significantly showed the synergistic interaction of 5-Aza and bortezomib inhibition in multiple myeloma. Moreover, CD9 transfected U266 cells had significantly increased bortezomib sensitivity suggested that one reason of the synergistic interaction of 5-Aza and bortezomib inhibition is CD9 expression.

From the CD9 functional study results, we found that CD9 expression did not affect the cell clone formation ability, cell cycle and the major cytokines (VEGF, IL-6 and IGF-1) secreted, but significantly affected the interaction beteen MM cells and their microenvironment. It significantly inhibited U266 cells' adhesion to HS-5 and also the primary bone marrow stromal cells, but CD9 expression increased U266 cells' adhesion to Fibronectin. MM cells localize within the BM through the interaction of adhesion receptors with their ligands on BM stromal cells and extracellular matrix proteins such as fibronectin [31]. It has been demonstrated that MM cells in the BM microenvironment are much less sensitive to chemotherapeutic agents [32], [33]. This phenomenon is termed "cell adhesion-mediated drug resistance" (CAM-DR) and it is thought to be one of the major mechanisms by which MM cells escape the cytotoxic effects of therapeutic agents. However, until now, despite extensive investigations [34], the adhesion molecules critical for CAM-DR in MM is poorly understood. Here, we found that CD9 could overcome the CAM-DR of bortezomib through inhibiting MM cells' adherence to stromal cells but not fibronectin. This is consistent with Niranjan's report that fibronectin adherence did not protect MM cells from tipifarnib- or bortezomib-induced apoptosis. Stroma cell adhered MM cells were partially protected relative to suspension cells, whereas fibronectin-adhered tumor cells seemed more sensitive to drug treatment [35].

Myeloma cell adhesion to BMSCs supports cell survival, proliferation, and cell adhesion-mediated drug resistance (CAM-DR) via signaling pathway activation, including the NF-kB (nuclear factor-kB), JAK/Stat3 (Janus kinase/signal transducer and activator of transcription-3), and MEK/MAPK (mitogen activated protein [MAP] kinase kinase/MAP kinase) pathways [36], [37]. Our gene-expression profiling results suggested that CD9 expression mainly affected the following three important signaling pathways respectively: the endoplasmic reticulum stress pathway, cell adhesion related signaling pathways and osteoclast differentiation related signaling pathway. The anti-cancer mechanisms of bortezomib elucidated by preclinical studies include induction of endoplasmic reticulum (ER) stress and pro-apoptotic Unfolded Protein Response (UPR) [38]. Activation of ER stress was further determined by checking several key proteins involving ER stress. CHOP and TRB3 expressed significantly higher in CD9 transfected U266 cells than in controls. During ER stress, the level of CHOP expression is elevated and CHOP functions to mediate programmed cell death [39]. TRB3 is also a stress-inducible nuclear protein, which has recently been shown to be involved in ER stress-induced apoptosis [40]. These data suggested that CD9 expression enhanced ER stress-mediated apoptosis by bortezomib in MM cells.

Our functional study results identified that CD9 really affected MM cells adhesion to the stromal cells and fibronectin. And this may be through cell adhesion related signaling pathways including upregulation of WASF1 and downregulation of MYL9, LMNA, CEECAM1, NCAM1, S100A6, PVRL4. Though mounting evidence has been shown that integrin-mediated cellular adhesion confers resistance to chemotherapy of multiple myeloma, our gene-expression profiling results didn't show that integrin as well as FAK expression changed. FAK is also an important mediator of focal adhesion formation and cell migration [41]. Then we checked some tyrosine-phosphorylated FAK protein expression using western blot. The results showed that only Phospho-FAK (Tyr925) expressed in cells and significantly increased in CD9 transfected U266 cells. Increased Phospho-FAK (Tyr925) was once reported to be correlated with loss of intercellular adhesion in breast cancer cells [42] and increased prostate cancer cell adhesion to fibronectin [43].

The gene-expression profiling results also show that CD9 expression may affect osteoclast differentiation in MM. Bone disease in patients with MM is characterized by increase in the numbers and activity of bone-resorpting osteoclasts and decrease in the number and function of bone-formation osteoblasts. CD9 may suppress osteoclastogenesis through up-regulation of TNFRSF1A, LILRB1, LILRB2 and down-regulation of CCL4, BLNK, LY96.

**Figure 6:**
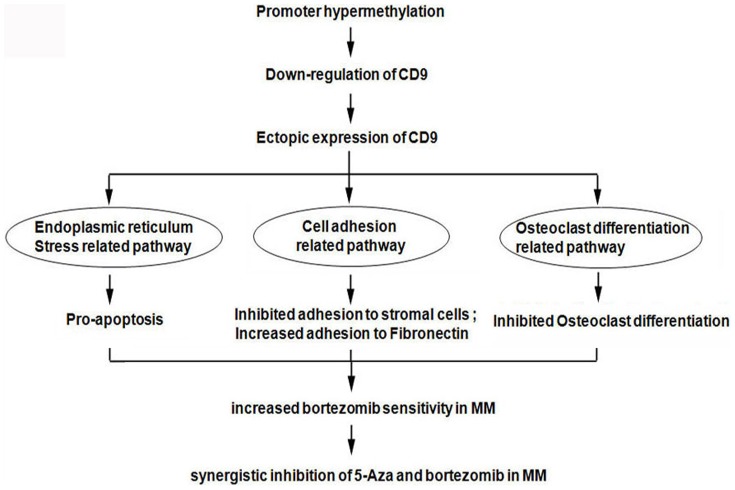
Diagram of the signaling pathways involved in CD9 expression's effect on bortezomib sensitivity in multiple myeloma.

In conclusions (Figure 6), we found that down-regulation of CD9 by methylation decreased bortezomib sensitivity in multiple myeloma. The mechanisms include three important signaling pathways: the endoplasmic reticulum stress pathway, cell adhesion related signaling pathway and osteoclast differentiation related signaling pathway. Combination therapy with de-methylation reagent 5-Aza may prove useful to the development of novel strategies for the treatment of bortezomib-resistant MM patients.

## Supporting Information

Figure S1 **Volcano Plot.** Differentially expressed genes with statistical significance were shown by Volcano Plot. The vertical lines correspond to 2.0-fold up and down and the horizontal line represents a P-value of 0.05. So the red point in the plot represents the differentially expressed mRNAs with statistical significance.
(TIF)

Table S1 **PCR primers used in this study.**
(DOCX)
