# Supplementary figures and images for: Down-Regulation of CD9 by Methylation Decreased Bortezomib Sensitivity in Multiple Myeloma
Source: PLoS One. 2014 May 2;9(5):e95765. doi: 10.1371/journal.pone.0095765 (PMC4008425; doi:10.1371/journal.pone.0095765)

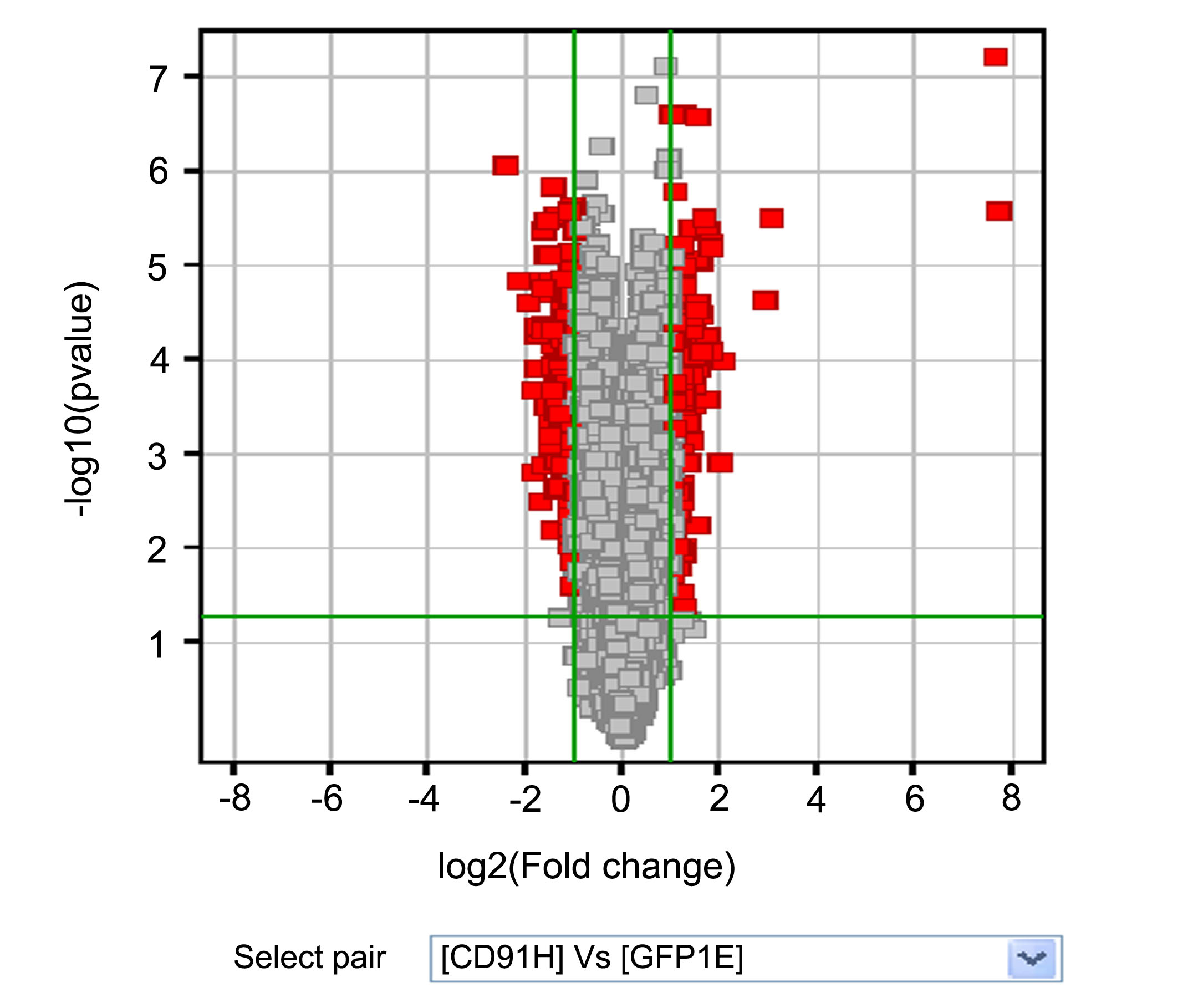

Supplement: Figure S1 — Volcano Plot. Differentially expressed genes with statistical significance were shown by Volcano Plot. The vertical lines correspond to 2.0-fold up and down and the horizontal line represents a P-value of 0.05. So the red point in the plot represents the differentially expressed mRNAs with statistical significance. (TIF) [file pone.0095765.s001.tif]
