# Supplementary material for: Down-Regulation of CD9 by Methylation Decreased Bortezomib Sensitivity in Multiple Myeloma
Source: PLoS One. 2014 May 2;9(5):e95765. doi: 10.1371/journal.pone.0095765 (PMC4008425; doi:10.1371/journal.pone.0095765)
Supplement: Table S1 — PCR primers used in this study. (DOCX) [file pone.0095765.s002.docx]

**Table S1. PCR primers used in this study**

| **primers** | **Primer sequences(5’-3’)** |
| --- | --- |
| **For determining *CD9* mRNA levels by RT-PCR** | |
| *CD9*-F | GATTGCTGTCCTTGCCATTGG |
| *CD9*-R | CTCATCCTTGTGGGAATATCC |
| **For detecting methylated *CD9* CGI by MSP** | |
| *CD9*-mF | AGAGGGTCGGGTTTCGC |
| *CD9*-mR | CACTCCCCCCGCCG |
| **For detecting unmethylated *CD9* CGI by MSP** | |
| *CD9*-uF | AGAGGGTTGGGTTTTGTGG |
| *CD9*-uR | CACTCCCCCCACCAACCA |
| **For detecting detail methylation status of *CD9* CGI by BGS** | |
| *CD9*-BGS1 | GGGAAATAGGGAGAGGGT |
| *CD9*-BGS2 | CTCACTCACCCAAAAAATA |
| **For standardizing the amount of RNA for RT-PCR** | |
| *GAPDH*-F | TCCTGTGGCATCCACGAAACT |
| *GAPDH*-R | GAAGCATTTGCGGTGGACGAT |
